# Supplementary material for: Molecular determinants of cardiac lymphatic dysfunction in a chronic pressure-overload model
Source: EMBO Mol Med. 2025 Dec 11;18(1):325–55. doi: 10.1038/s44321-025-00345-w (PMC12808729; doi:10.1038/s44321-025-00345-w)
Supplement: Supplementary file 1 — Appendix [file 44321_2025_345_MOESM1_ESM.pdf]

Table of content Appendix

Appendix Fig. S1 Cardiac LEC expression profiles in AngII model 2
Appendix Table S1 Mean transcripts and genes per cell 3
Appendix Table S2 Antibodies and reagents used for FACS in mouse.......................... 4
Appendix Table S3 Antibodies and reagents used in tissue sections 5
Appendix Table S4 Antibodies and reagents used for whole mount staining 6

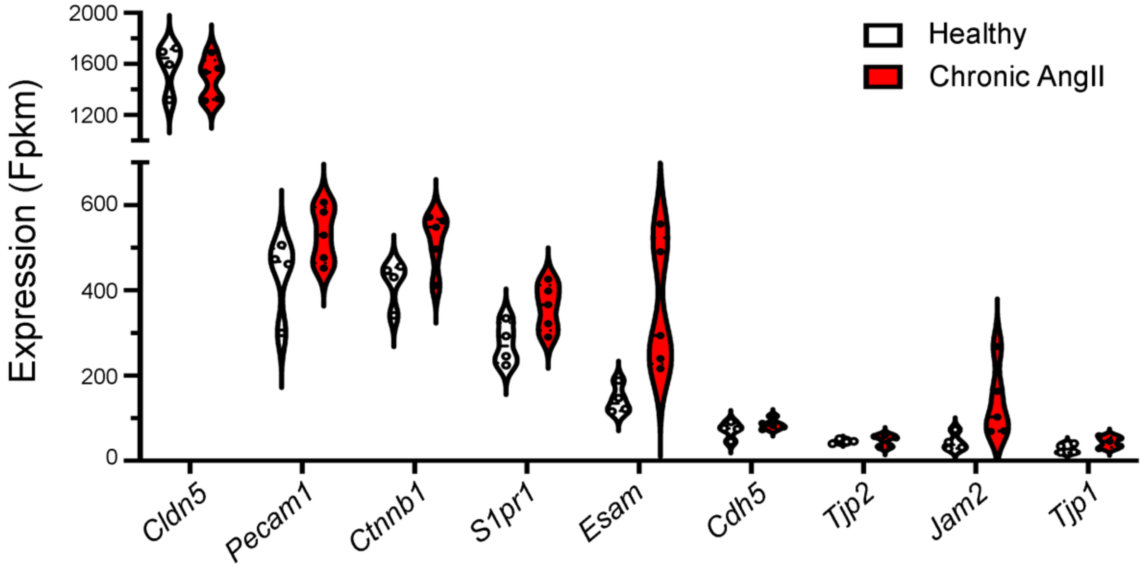

Appendix Fig. S1 Cardiac LEC expression profiles in AngII model

Expression level [*Fpkm counts*] changes in vascular barrier-related genes in cardiac LECs isolated for bulk RNAseq (n=4-5 samples/group) following pressure-overload induced by chronic AngII infusion for 6 weeks in C57 mice (adapted from *Song et al*. GSE150041).

Appendix Table S1 Mean transcripts and genes per cell

**BALB/c ECs**	***N* (cells)**	**Σ nUMI (reads)**	**Σ nGene**	**nUMI / nGene**
Healthy LEC	*402*	3079	1400	2,2
Healthy BEC	*1196*	2514	1324	1,9
Healthy vBEC	*216*	3002	1323	2,3
**Global healthy**	***1814***	**2699**	**1341**	**2,0**
TAC LEC	*453*	5027	2075	2,4
TAC BEC	*833*	3006	1468	2,0
TAC vBEC	*168*	6811	2338	2,9
**Global TAC**	***1459***	**4096**	**1761**	**2,3**
**C57 ECs**	*N* (cells)	Σ nUMI (reads)	Σ nGene	nUMI / nGene
Healthy LEC	*150*	4023	1837	2,2
Healthy BEC	*380*	3008	1470	2,0
Healthy vBEC	*104*	5018	2101	2,4
**Global healthy**	***634***	**3564**	**1656**	**2,2**
TAC LEC	*81*	7606	2693	2,8
TAC BEC	*704*	4712	1992	2,4
TAC vBEC	*107*	8006	2733	2,9
**Global TAC**	***892***	**5370**	**2145**	**2,5**

Average Read counts and Features per cell in global EC population or main cluster

Appendix Table S2 Antibodies and reagents used for FACS in mouse

Antigen	Fluorochrome	host	Source	article
**CD16/32 (Fc block)**	-	rat	BD Pharmingen	553142
**Fixable Viability Dye (L/D)**	eFluor™ 455UV	-	ebioscience	65-0868-14
**CD31**	APC	rat	BD Pharmingen	551262
**CD45**	PerCP	rat	Sony Biotechnology	1115650
**Lyve1**	PE	rat	RnD systems	FAB212SP
**Podoplanin**	A488	hamster	Sony Biotechnology	1320070
**Podoplanin**	A488	hamster	Ozyme/Biolegend	127406

Appendix Table S3 Antibodies and reagents used in tissue sections

antigen	article	supplier	species reactivity	host	dilution	Working conc (µg/mL)
**alpha SMA-FITC**	*F3777*	Sigma Aldrich	mouse	mouse	1/100	**28**
**CD31/PECAM**	*553371*	BD	mouse	rat	1/100	**0.6**
**CD68**	*14-0681-82*	eBioscience	mouse	rat	1/800	**5**
**CCL21**	*AF457*	RnD systems	mouse	goat	1/100	**5**
**Claudin-5**	*34-1600*	Invitrogen	mouse	rabbit	1/400	**0.6**
**F4/80**	*MCA497R*	Abd Serotec	mouse	rat	1/200	**5**
**LYVE-1**	*103-PA50*	Reliatech	mouse	rabbit	1/1000	**0.4**
**Biotinylated LYVE-1**	ALY7 13-0443-82	eBiosciences	mouse	rat	1/500	**0.5**
**CD206/MRC1**	*ab64693*	Abcam	mouse	rabbit	1/2500	**4**
**Pdl1**	124302	Biolegend	mouse	rat	1/50	**10**
**Podoplanin**	*14-5381-82*	eBioscience	mouse	hamster	1/10 000	**1**
**Reelin**	*AF3820*	RnD systems	mouse	goat	1/50	**4**
**VEGF-C**	*ab9546*	Abcam	mouse	rabbit	1/500	**2**
**VCAM-1**	*sc-19982*	SantaCruz	mouse	rat	1/500	**0.4**
**ICAM-1**	*14-0541-82*	eBioscience	mouse	rat	1/100	**5**
**VE-cadherin**	*AF1002*	RnD systems	mouse	goat	1/200	**1**
**WGA**	*FP-CE8070*	Interchim			1/100	**1**

reactivity	article	supplier	Fluorochrome	Working conc (µg/ mL)
**Donkey anti-Rat**	*712-545-153*	Jackson Immunoresearch	**AF488**	**3**
**Donkey anti-Rat**	*712-166-153*	Jackson Immunoresearch	**Cy3**	**3**
**Goat anti-Rat**	*A-21247*	Thermo Fisher Scientific/ Invitrogen	**AF647**	**0.8**
**Donkey anti-Rabbit**	*711-165-152*	Jackson Immunoresearch	**Cy3**	**3**
**Donkey anti-Rabbit**	*711-605-152*	Jackson Immunoresearch	**AF647**	**1.5**
**Donkey anti-Goat**	*A50-201D2*	Interchim	**DYLIGHT488**	**1.3**
**Donkey anti-Goat**	*A50-201D3*	Interchim	**DYLIGHT 550**	**1.3**
**Streptavidin**	*FP-CA5570*	Interchim	**Fluoprobe 547**	**0.7**
**Streptavidin**	*FP-CA5640*	Interchim	**Fluoprobe 647**	**0.7**
**Goat anti-Hamster**	*A-21110*	Thermo Fisher Scientific / Invitrogen	**AF488**	**0.8**

Appendix Table S4 Antibodies and reagents used for whole mount staining

antigen	article	supplier	species reactivity	host	dilution	Conc (µg/mL)
**alpha SMA-Cy3**	*C6198*	Sigma Aldrich	mouse	mouse	1/500	**3**
**CCL21**	*AF457*	RnD systems	mouse	goat	1/100	**5**
**LYVE-1**	*103-PA50AG*	Reliatech	mouse	rabbit	1/500	**0.8**
**Biotinylated LYVE1**	ALY7 13-0443-82	eBiosciences	mouse	rat	1/500	**0.5**
**Podocalyxin**	AF1556	RnD systems	mouse	goat	1/200	**1**
**Podoplanin**	*14-5381-82*	eBioscience	mouse	hamster	1/500	**0.1**
**antigen**	article	supplier	Fluoro-chrome		dilution	Conc (µg/ mL)
**Donkey anti-Rabbit**	*711-165-152*	Jackson Immunoresearch	**Cy3**		1/500	**3**
**Donkey anti-Rabbit**	*711-605-152*	Jackson Immunoresearch	**AF647**		1/500	**3**
**Donkey anti-Goat**	*A50-201D3*	Interchim	**DYLIGHT550**		1/500	**3**
**Donkey anti goat**	*705-585-147*	Jackson Immunoresearch	**Cy3**		1/400	**1.25**
**Streptavidin**	*FP-CA5570*	Interchim	**Fluoprobe547**		1/300	**0.7**
**Goat anti-Hamster**	*A21113*	Thermo Fisher Scientific / Invitrogen	**Cy3**		1/1000	**0.8**
