# Supplementary material for: Molecular determinants of cardiac lymphatic dysfunction in a chronic pressure-overload model
Source: EMBO Mol Med. 2025 Dec 11;18(1):325–55. doi: 10.1038/s44321-025-00345-w (PMC12808729; doi:10.1038/s44321-025-00345-w)
Supplement: Supplementary file 15 — Movie EV1 [file 44321_2025_345_MOESM15_ESM.zip › movie EV1/Movie EV1 legend.docx]

### **Movie EV1 Lymphatic CCL21 expression in healthy hearts [BALB/c]**

Visualization of Ccl21 chemokine (magenta) expression in lymphatic capillaries stained for Lyve (cyan). Scalebar 50 µm.
