# Supplementary material for: Molecular determinants of cardiac lymphatic dysfunction in a chronic pressure-overload model
Source: EMBO Mol Med. 2025 Dec 11;18(1):325–55. doi: 10.1038/s44321-025-00345-w (PMC12808729; doi:10.1038/s44321-025-00345-w)
Supplement: Supplementary file 16 — Movie EV2 [file 44321_2025_345_MOESM16_ESM.zip › movie EV2/Movie EV2 legend.docx]

### **Movie EV2 Lymphatic valves in healthy hearts [C57]**

Lymphatic valves, and blood vessel capillaries, stained with Podocalyxin (blue) and lymphatic capillaries with Lyve (shown in green or light pink). Valves highlighted with red dots. Scalebar 200 µm.
