# Supplementary figures and images for: Molecular determinants of cardiac lymphatic dysfunction in a chronic pressure-overload model
Source: EMBO Mol Med. 2025 Dec 11;18(1):325–55. doi: 10.1038/s44321-025-00345-w (PMC12808729; doi:10.1038/s44321-025-00345-w)

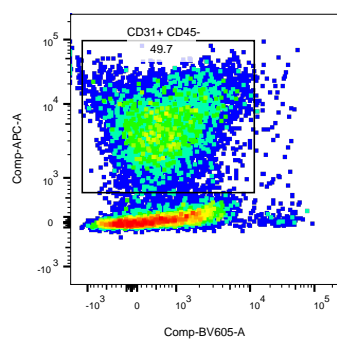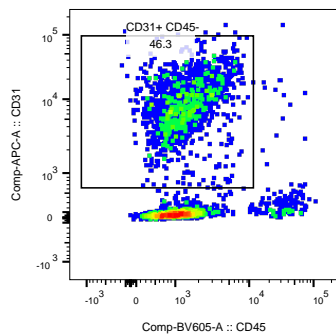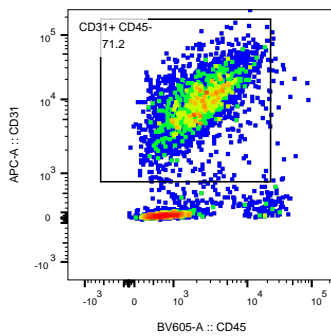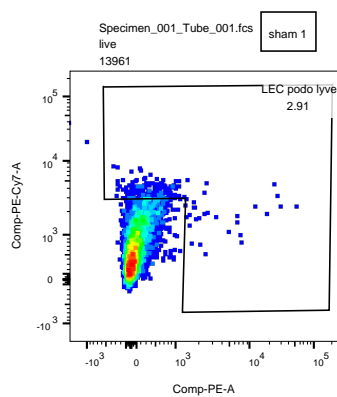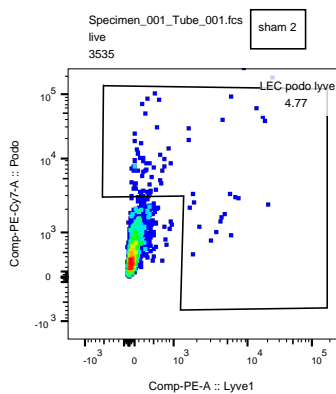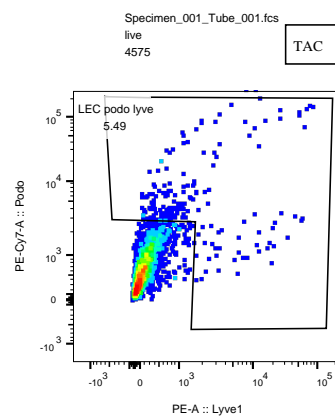

Specimen\_001\_Tube\_001.fcs  
CD31+ CD45-  
6943

Specimen\_001\_Tube\_001.fcs  
CD31+ CD45-  
1636

Specimen\_001\_Tube\_001.fcs  
CD31+ CD45-  
3258

Supplement: Supplementary file 17 — Source data Fig. 1 [file 44321_2025_345_MOESM17_ESM.zip › Fig 1/analysis scRNAsort2021 Fig. 1a.pdf]

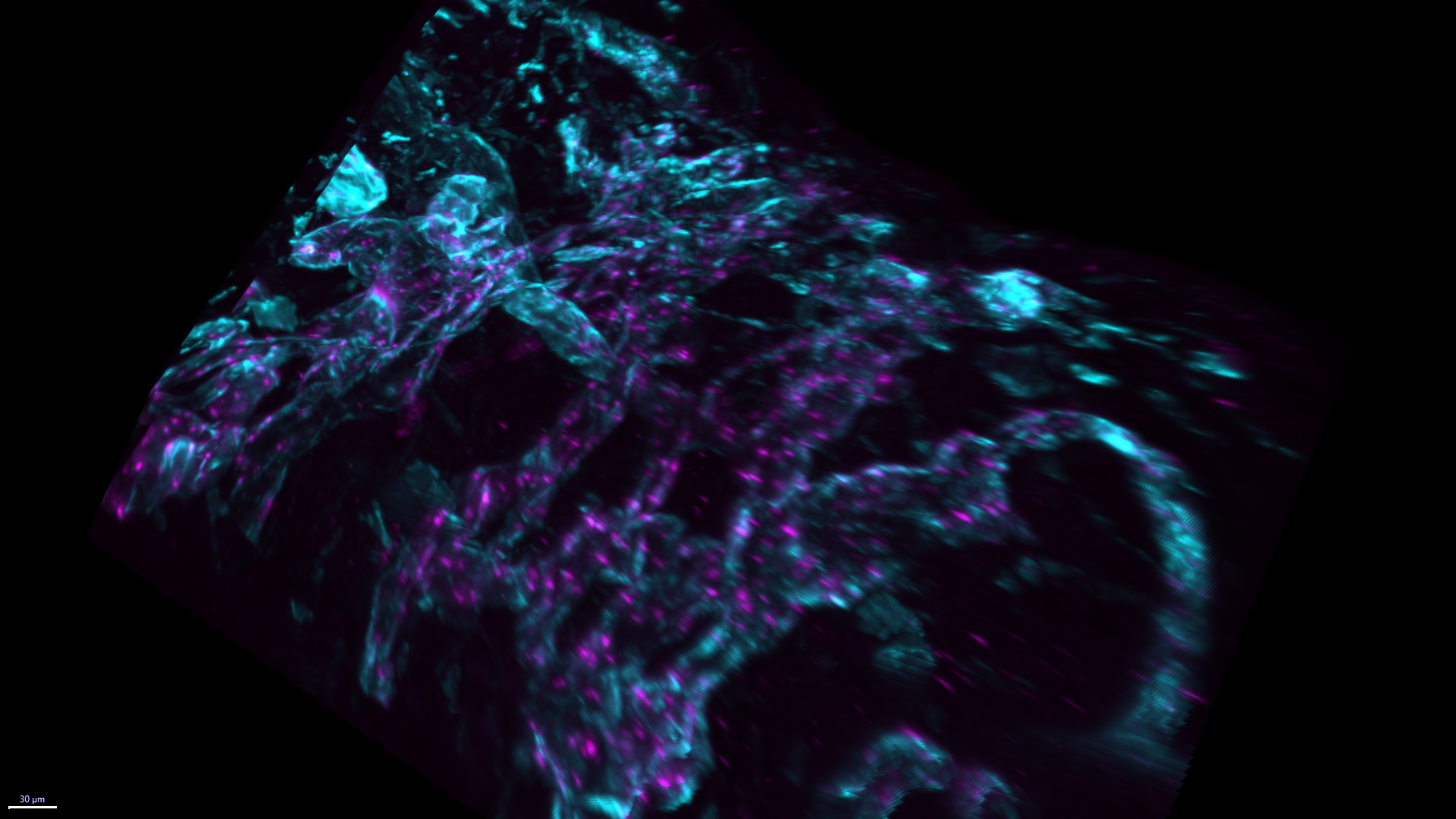

Supplement: Supplementary file 18 — Source data Fig. 2 [file 44321_2025_345_MOESM18_ESM.zip › Fig 2/2F-1.tif]

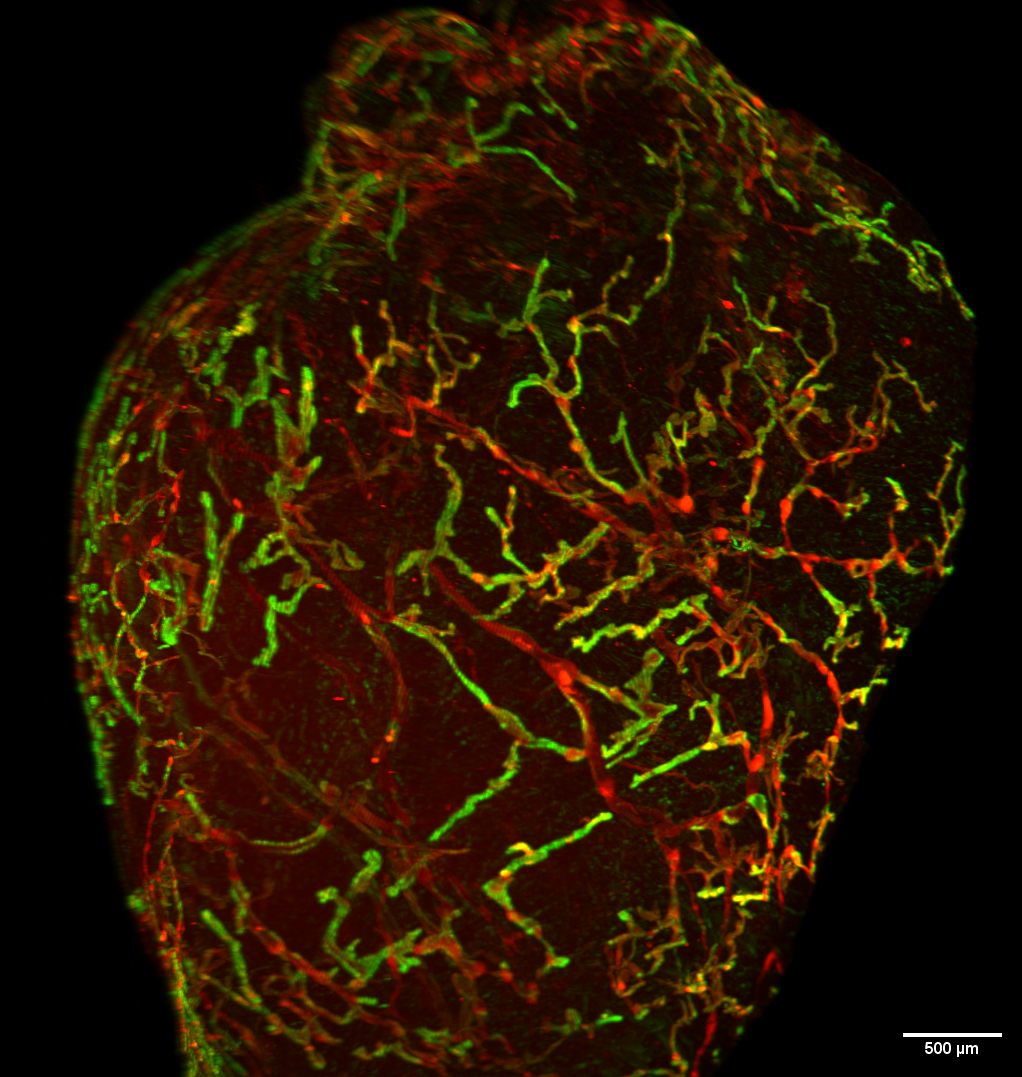

Supplement: Supplementary file 18 — Source data Fig. 2 [file 44321_2025_345_MOESM18_ESM.zip › Fig 2/2F-2.tif]

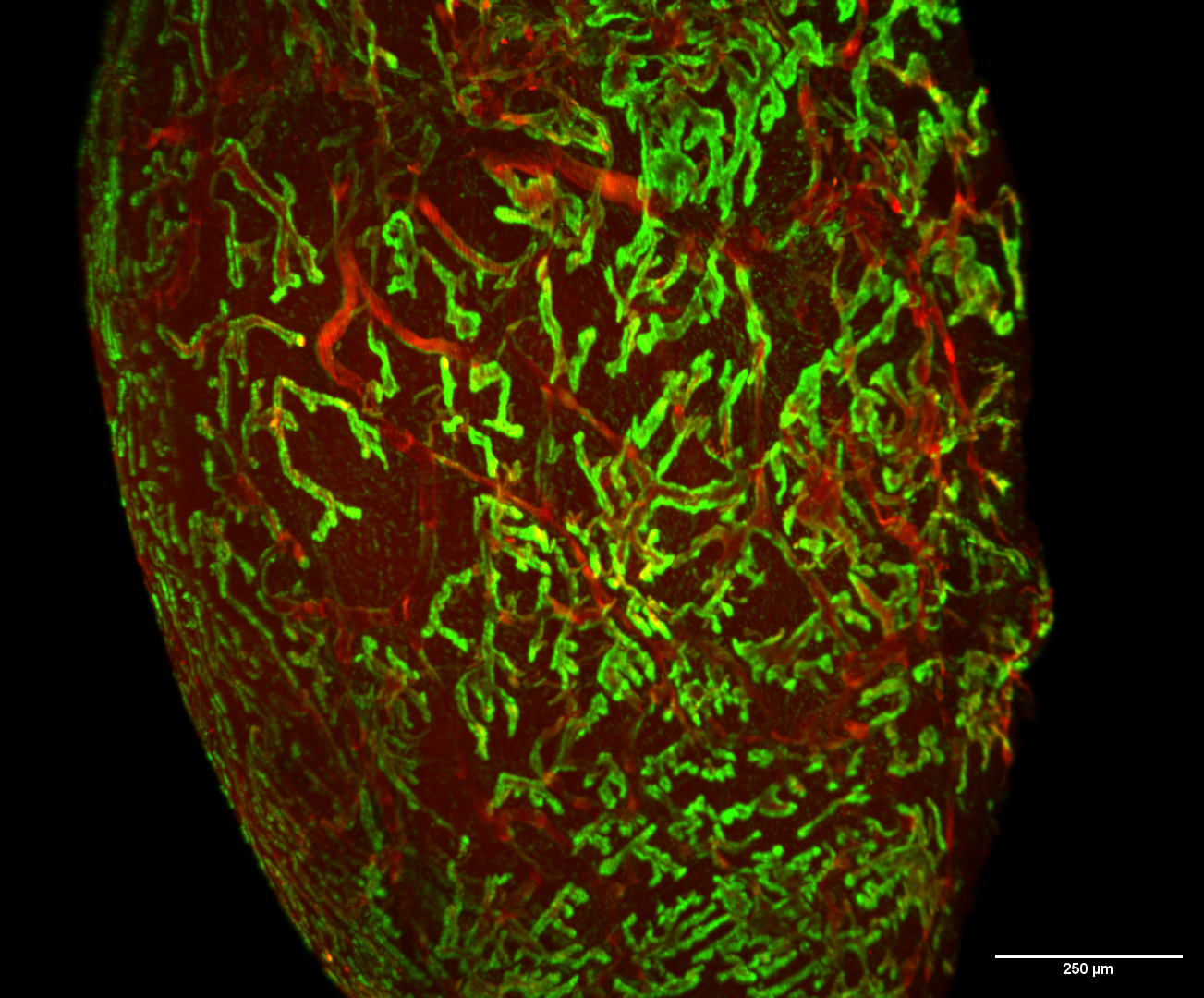

Supplement: Supplementary file 19 — Source data Fig. 3 [file 44321_2025_345_MOESM19_ESM.zip › Fig 3/3G-2.tif]

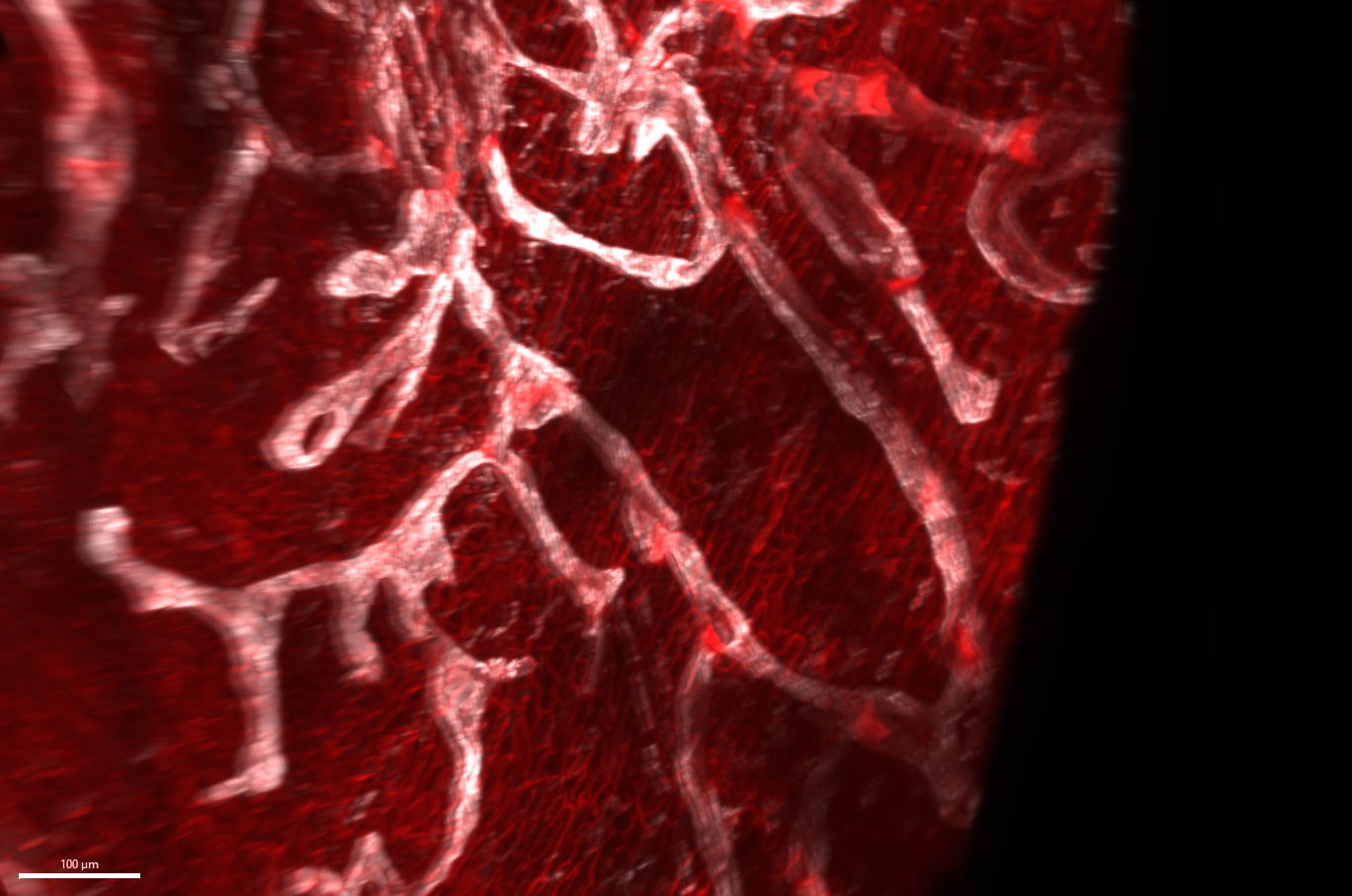

Supplement: Supplementary file 20 — Source data Fig. 4 [file 44321_2025_345_MOESM20_ESM.zip › Fig 4/4D-1.tif]

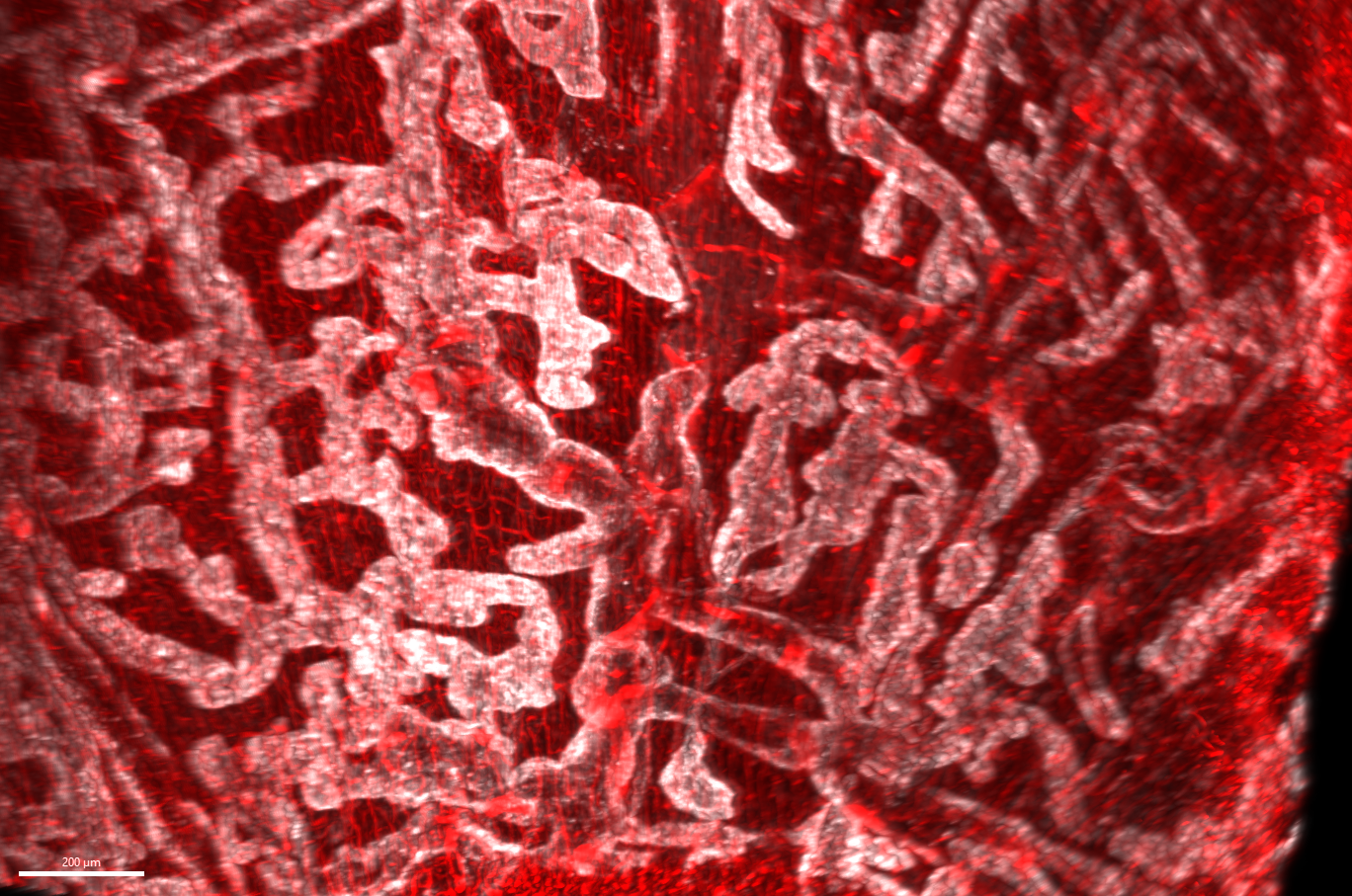

Supplement: Supplementary file 20 — Source data Fig. 4 [file 44321_2025_345_MOESM20_ESM.zip › Fig 4/4D-2.tif]

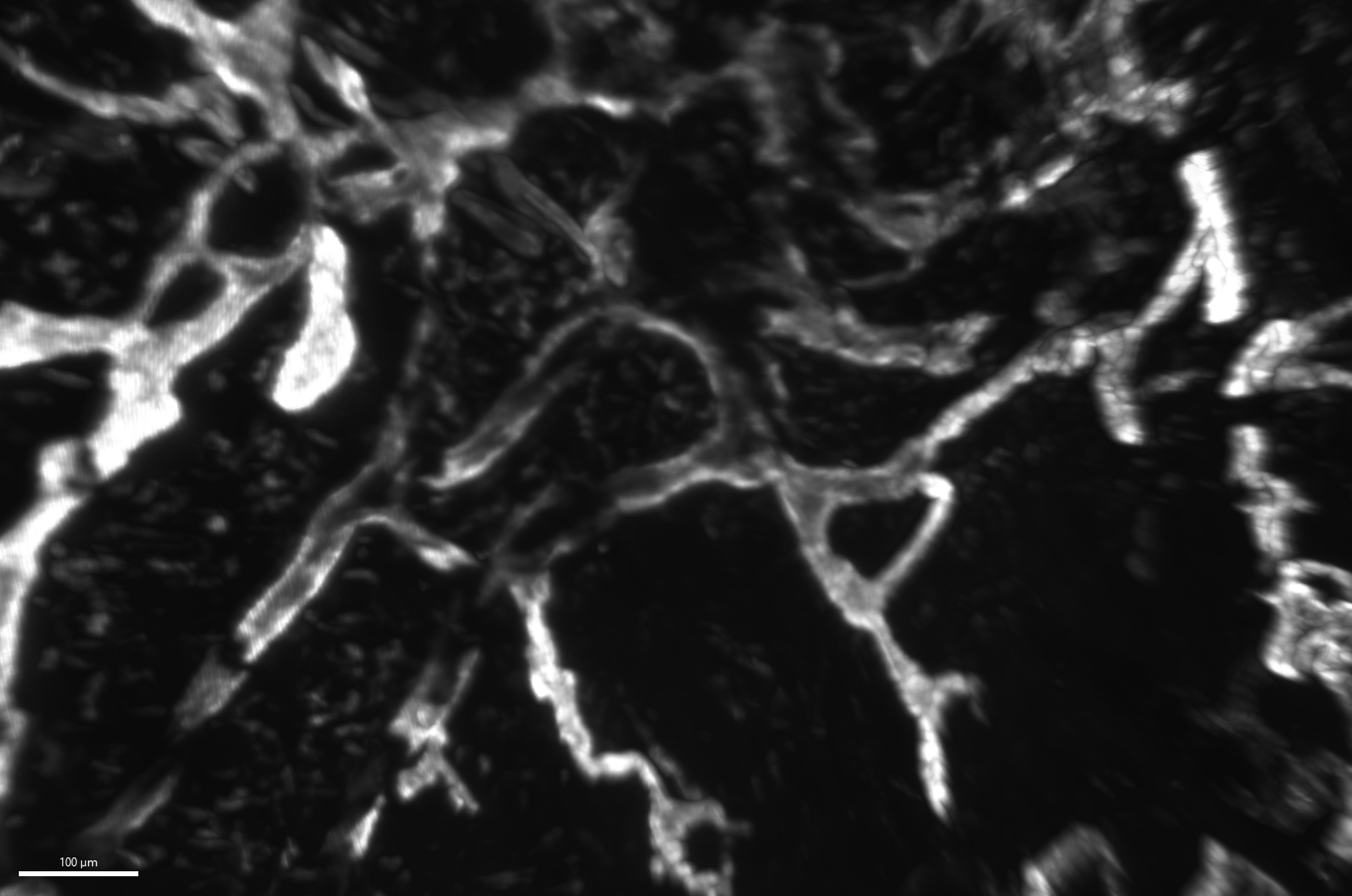

Supplement: Supplementary file 21 — Source data Fig. 5 [file 44321_2025_345_MOESM21_ESM.zip › Fig 5/5H-1A.tif.tif]

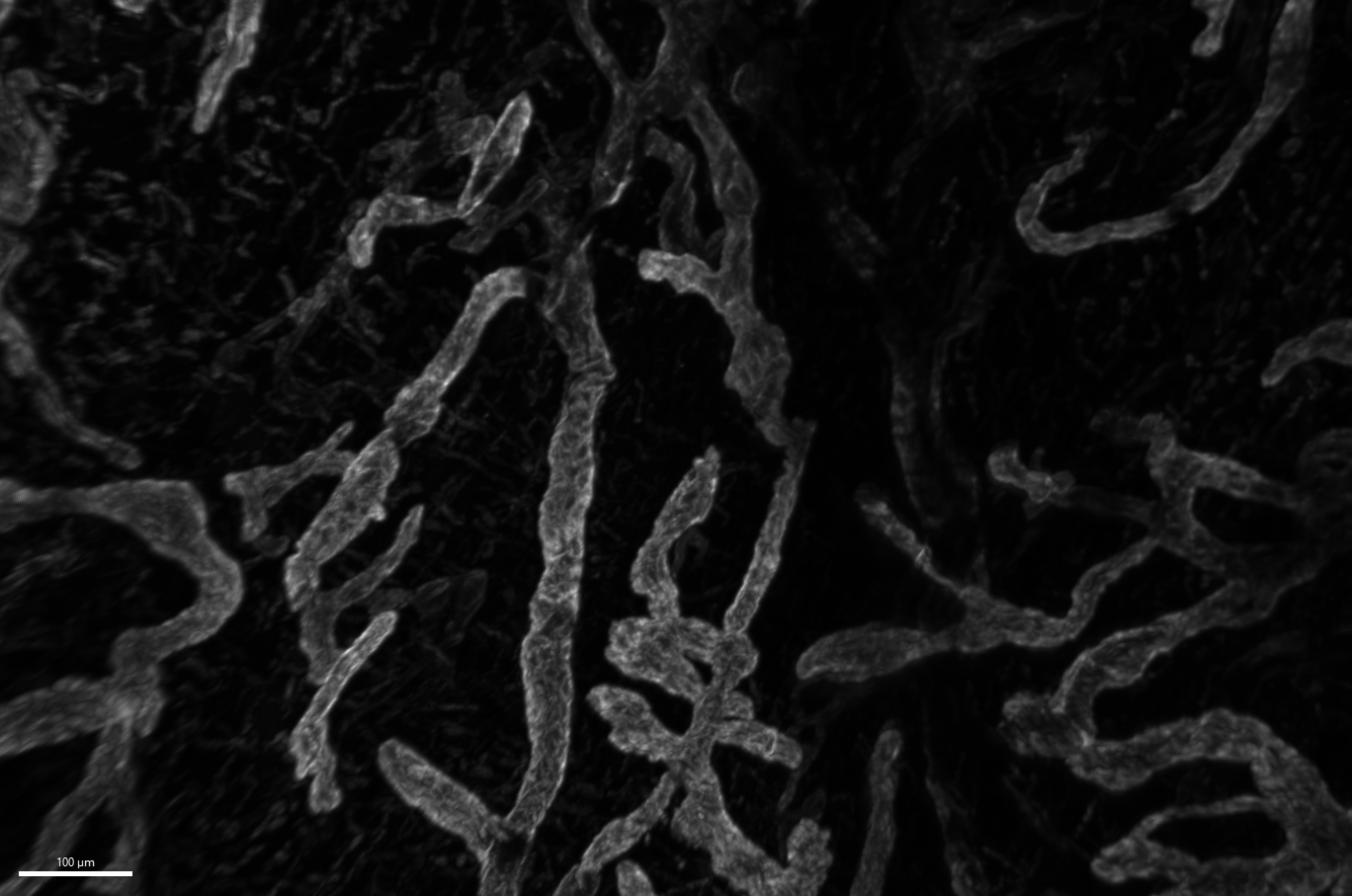

Supplement: Supplementary file 21 — Source data Fig. 5 [file 44321_2025_345_MOESM21_ESM.zip › Fig 5/5H-2A.tif.tif]

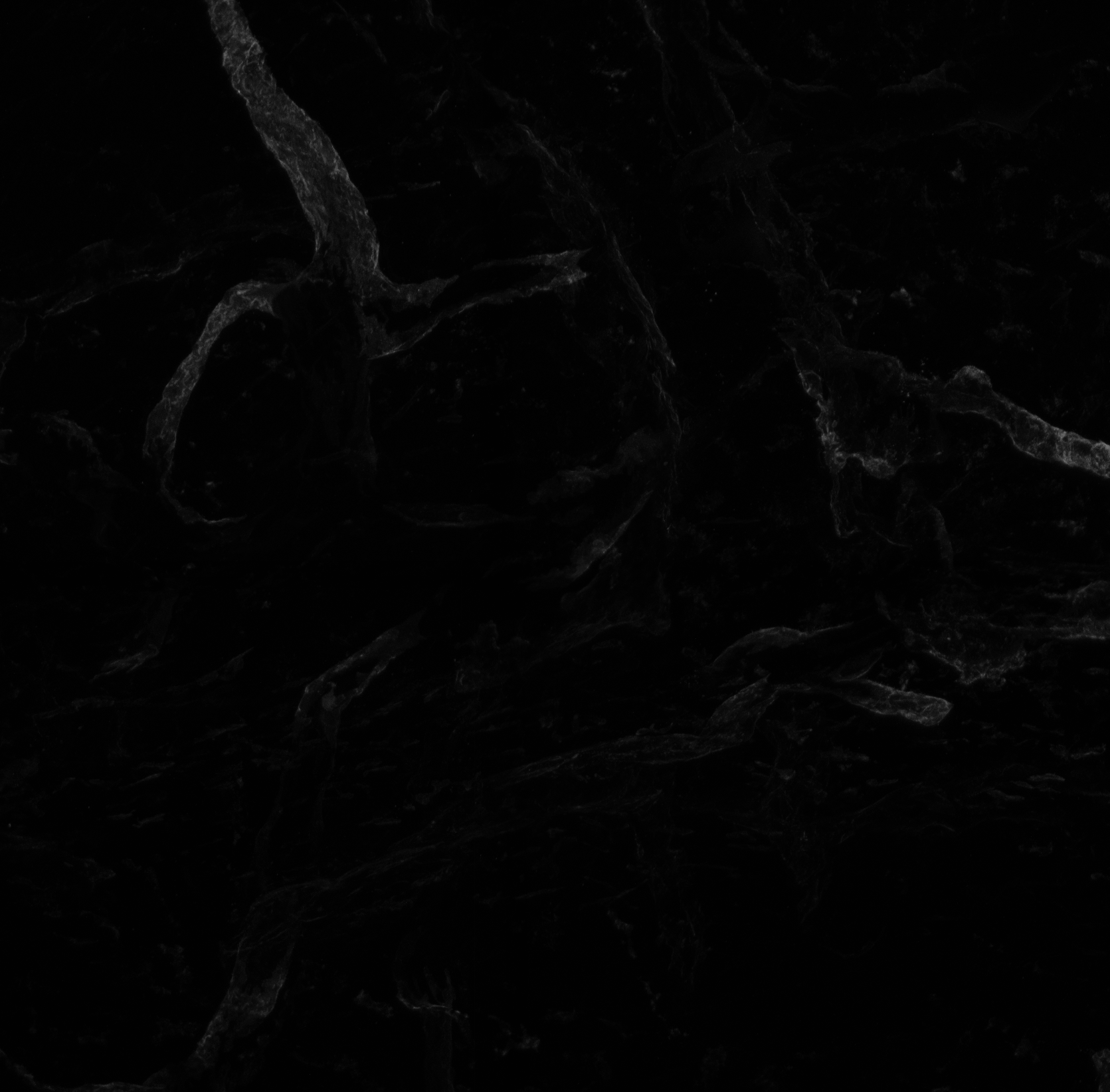

Supplement: Supplementary file 22 — Source data Fig. 6 [file 44321_2025_345_MOESM22_ESM.zip › Fig 6/panel A/6A-1.tif]

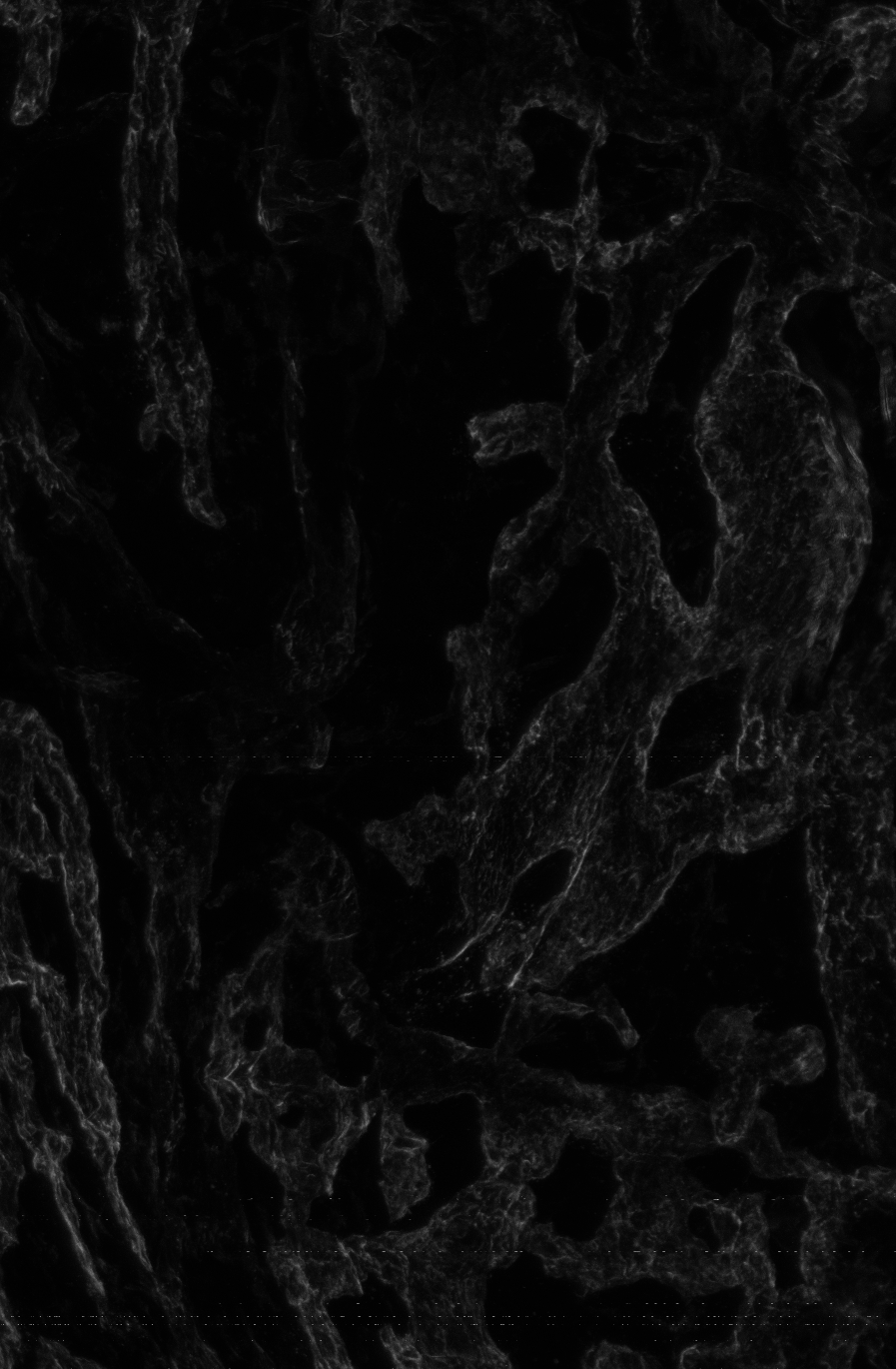

Supplement: Supplementary file 22 — Source data Fig. 6 [file 44321_2025_345_MOESM22_ESM.zip › Fig 6/panel A/6A-2.tif]

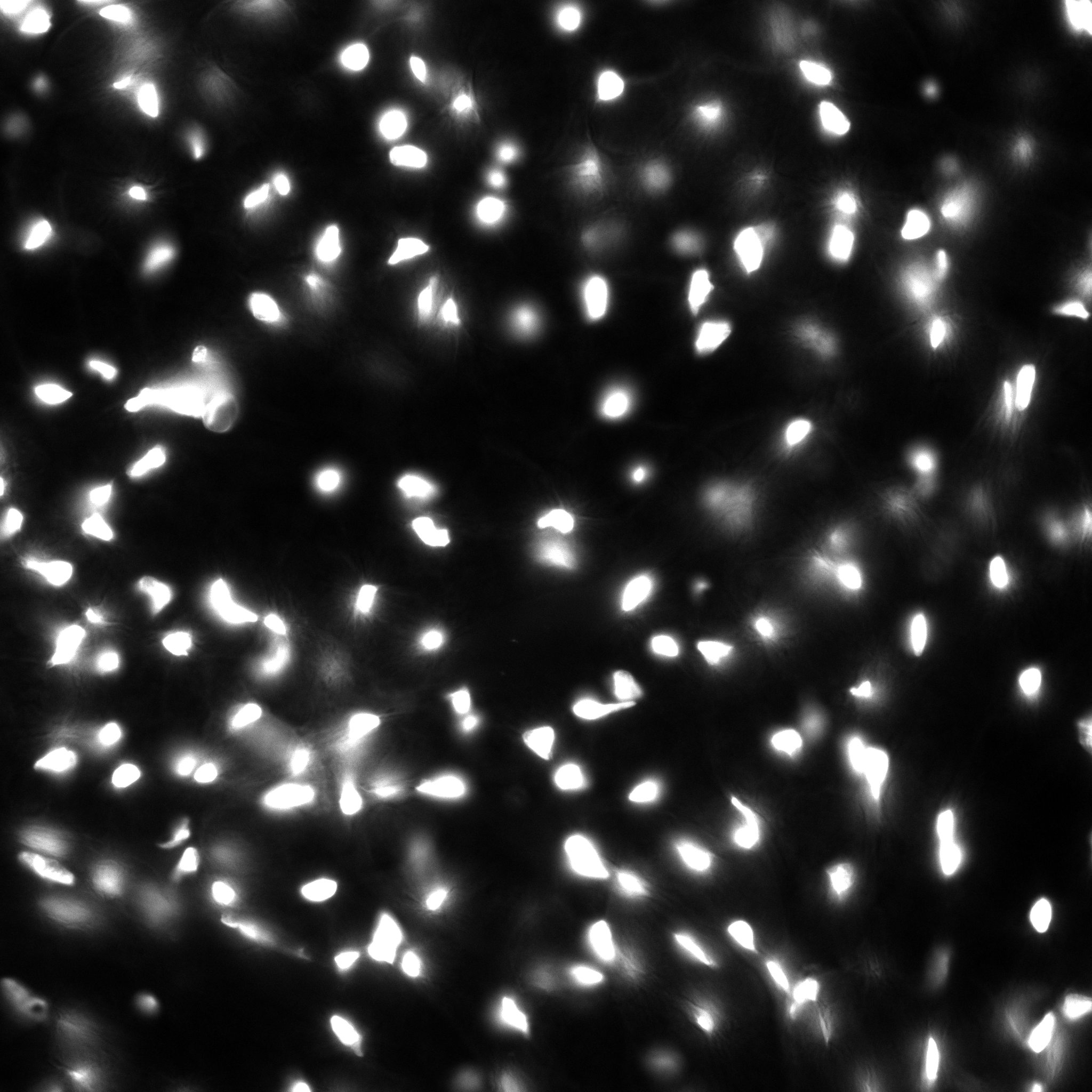

Supplement: Supplementary file 22 — Source data Fig. 6 [file 44321_2025_345_MOESM22_ESM.zip › Fig 6/panel C/6C-1.tif]

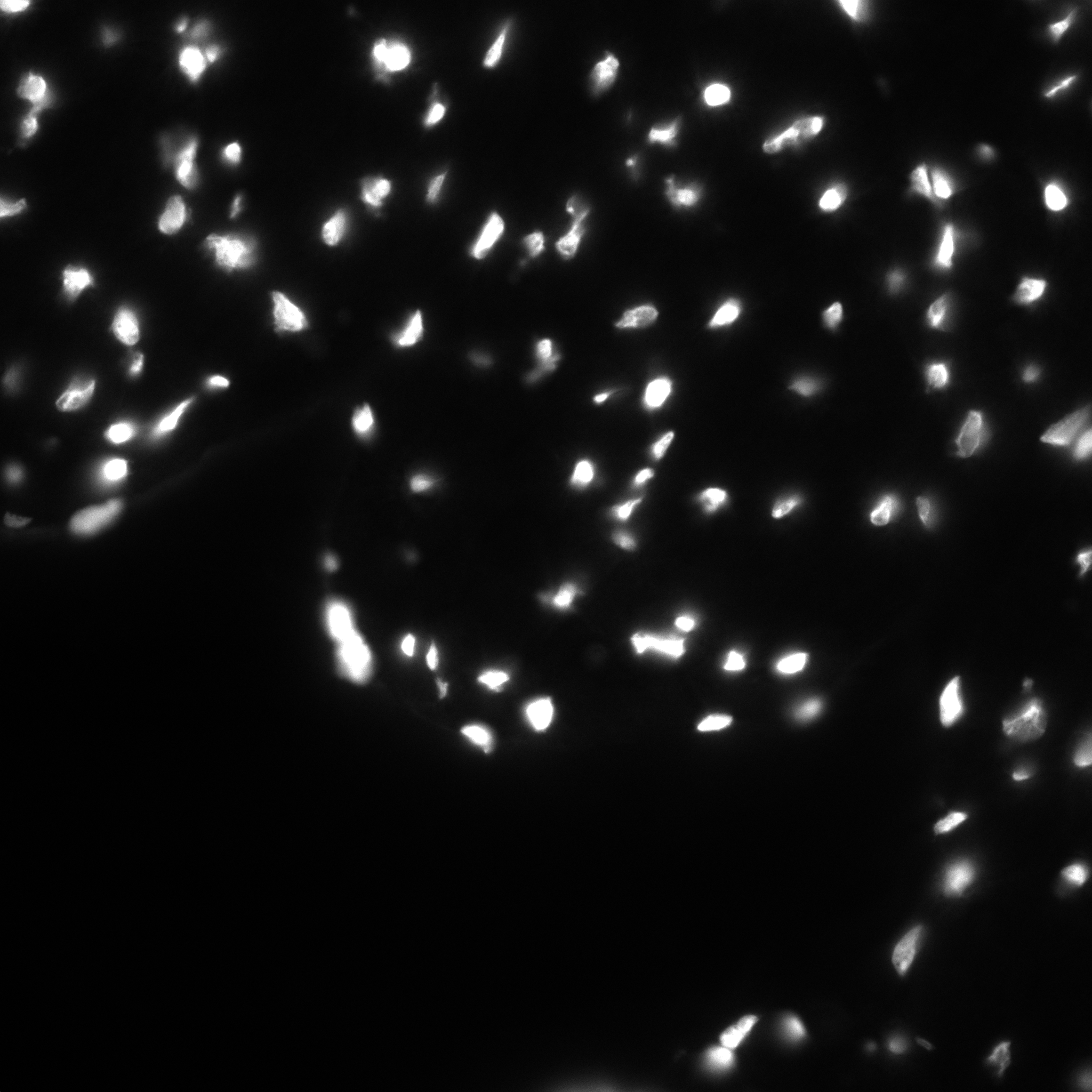

Supplement: Supplementary file 22 — Source data Fig. 6 [file 44321_2025_345_MOESM22_ESM.zip › Fig 6/panel C/6C-2.tif]
