# Supplementary material for: Molecular determinants of cardiac lymphatic dysfunction in a chronic pressure-overload model
Source: EMBO Mol Med. 2025 Dec 11;18(1):325–55. doi: 10.1038/s44321-025-00345-w (PMC12808729; doi:10.1038/s44321-025-00345-w)
Supplement: Supplementary file 22 — Source data Fig. 6 [file 44321_2025_345_MOESM22_ESM.zip › Fig 6/panel B/analysis scRNAsort2021 Fig 6b.pdf]

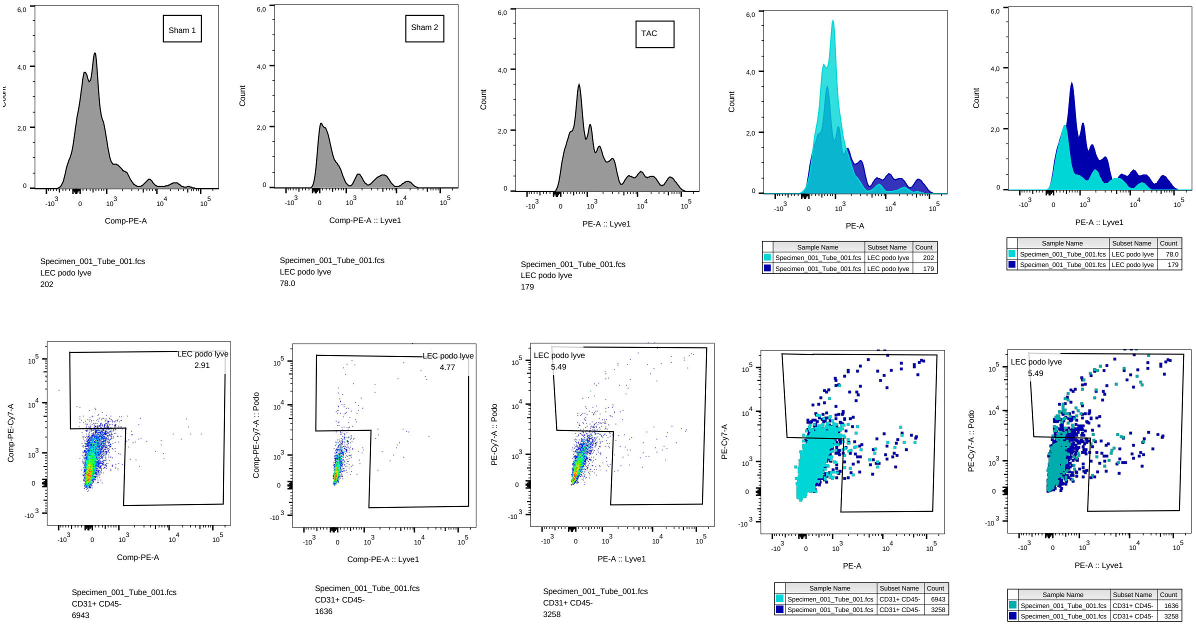

	Sample Name	Subset Name	Count
	Specimen_001_Tube_001.fcs	LEC podo lyve	202
	Specimen_001_Tube_001.fcs	LEC podo lyve	179

	Sample Name	Subset Name	Count
	Specimen_001_Tube_001.fcs	LEC podo lyve	78.0
	Specimen_001_Tube_001.fcs	LEC podo lyve	179

	Sample Name	Subset Name	Count
	Specimen_001_Tube_001.fcs	CD31+ CD45-	6943
	Specimen_001_Tube_001.fcs	CD31+ CD45-	3258

	Sample Name	Subset Name	Count
	Specimen_001_Tube_001.fcs	CD31+ CD45-	1636
	Specimen_001_Tube_001.fcs	CD31+ CD45-	3258
