# Supplementary material for: Molecular determinants of cardiac lymphatic dysfunction in a chronic pressure-overload model
Source: EMBO Mol Med. 2025 Dec 11;18(1):325–55. doi: 10.1038/s44321-025-00345-w (PMC12808729; doi:10.1038/s44321-025-00345-w)
Supplement: Supplementary file 23 — Expanded View Figures [file 44321_2025_345_MOESM23_ESM.pdf]

Expanded View Figures

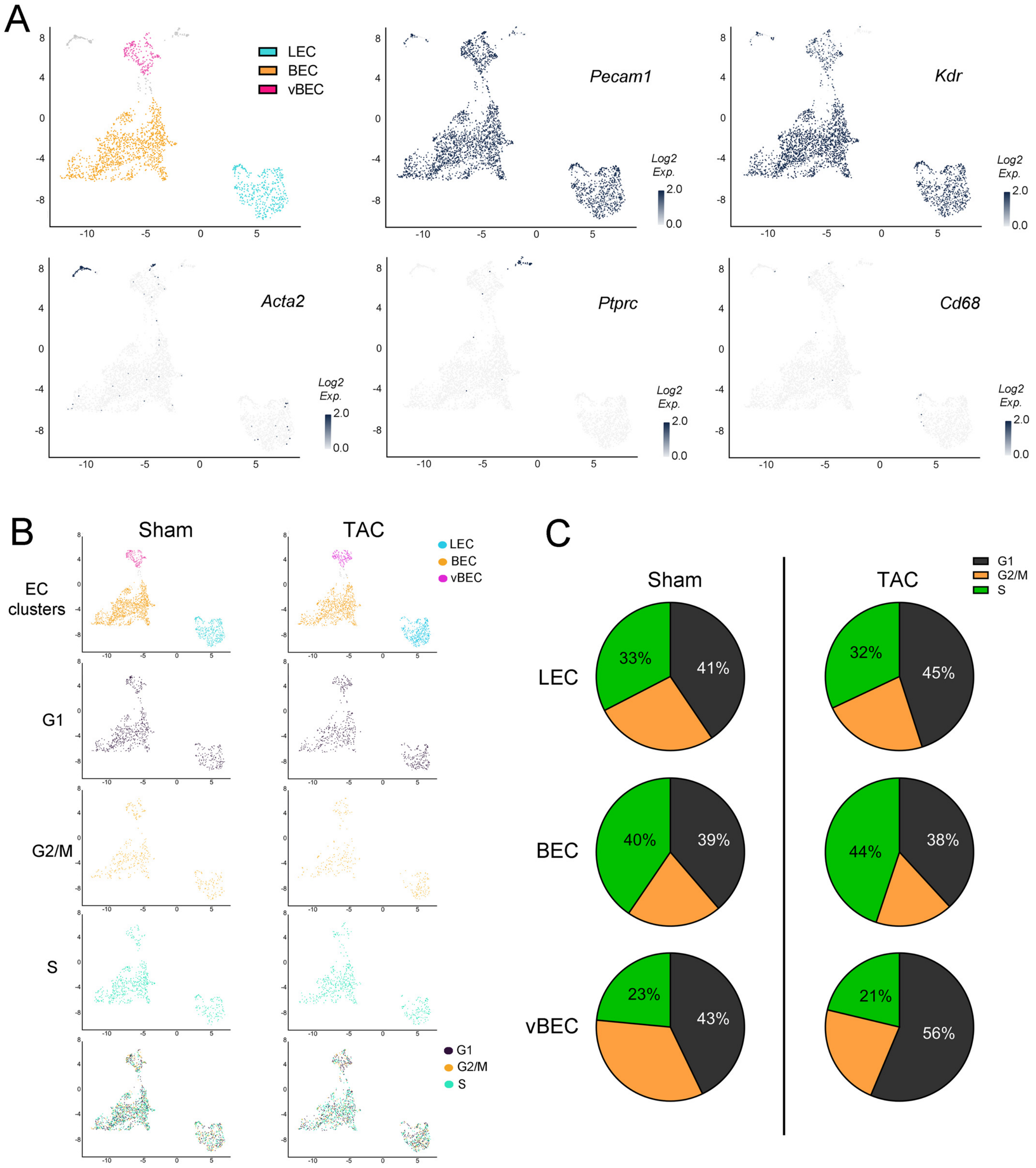

Figure EV1. Marker genes and cycle phase distribution of cardiac endothelial cells.

(**A**) Visualization of cardiac EC clusters from healthy BALB/c mice ($n = 20$). The three main EC clusters expressed vascular markers (*Pecam1, Kdr*), but not immune (*Ptprc, Cd68*) or mural cell markers (*Acta2*). Expression levels shown as *Log2 normalized read counts*. (**B**, **C**) Visualization and quantification of CellLoupe-based scoring of cell cycle phases in main EC clusters from healthy and post-TAC BALB/c hearts.

 © The Author(s)

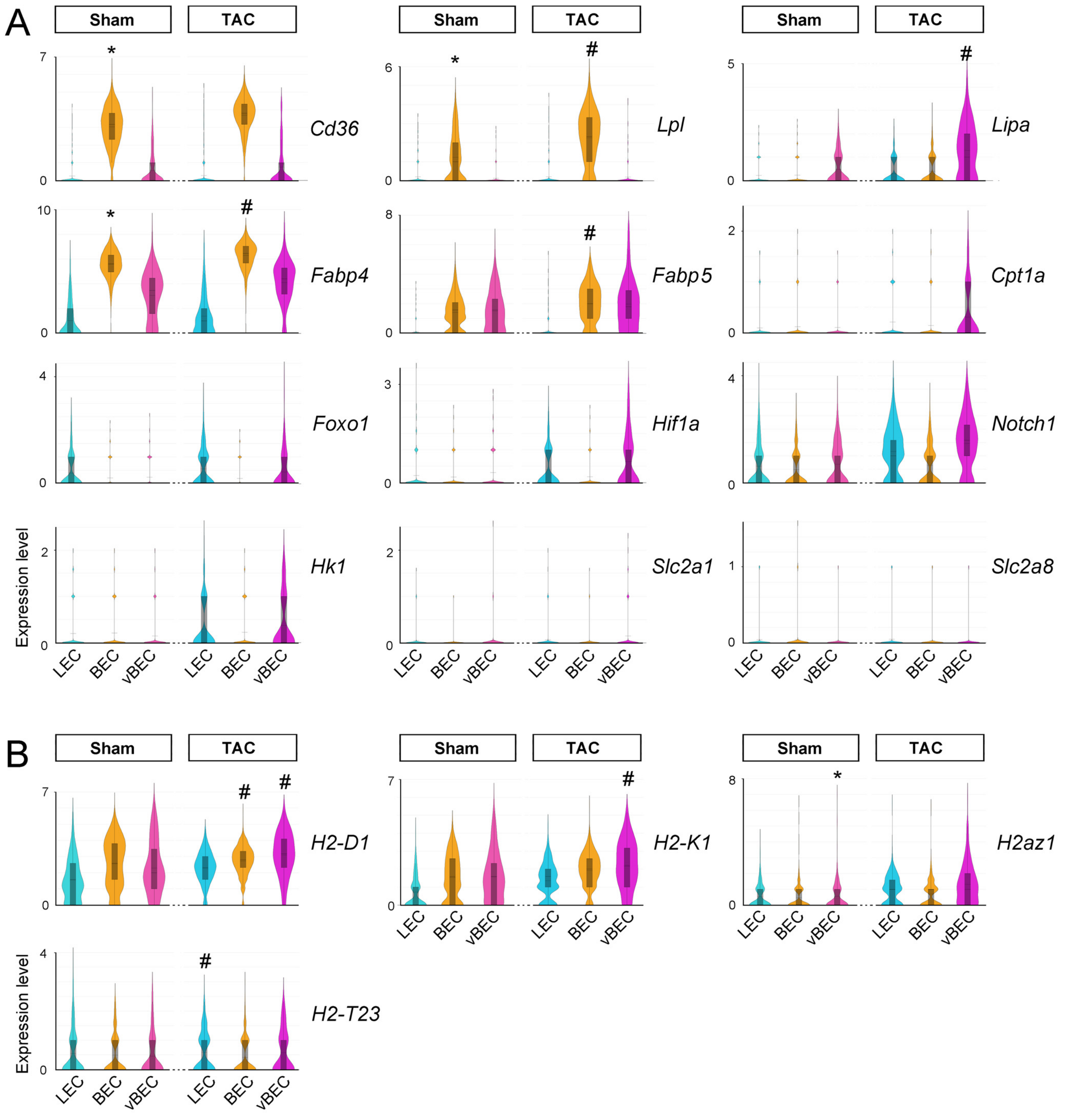

Figure EV2. Distinguishing EC markers related to metabolic and MHC class I genes.

(**A**) Examples of expression levels (*Log2 normalized read counts*) of genes involved in regulation of metabolism, including lipolysis (*Cd36, Lpl, Lpa, Fabp4, Fabp5, Cpt1a*) and glycolysis (*Foxo1, Hif1a, Notch, Hk1, Slc2a1, Slc2a8*). (**B**) Comparison of gene expression levels of main MHC class I molecules for antigen presentation in cardiac EC clusters from healthy ($n = 20$) and post-TAC ($n = 10$) BALB/c mice. Clusters significantly enriched for a given gene are denoted (*). For a full list of marker genes of EC clusters in healthy hearts, and exact *P* values, see Dataset EV1. Significantly altered genes post-TAC are denoted (#). For full lists of DEGs post-TAC see Datasets EV4 (LEC), EV5 (BEC), and EV6 (vBEC). *Cpt1a*, Carnitine palmitoyl transferase; *Fabp*, Fatty acid binding protein; *Foxo1*, Forkhead box protein O; *Hif1a*, Hypoxia-inducible factor; *Hk1*, Hexokinase-1; *Lpl*, Lipoprotein Lipase; *Lpa*, Lipase A; *Slc2a1*, Glut1; *Slc2a8*, Glut8.

© The Author(s)

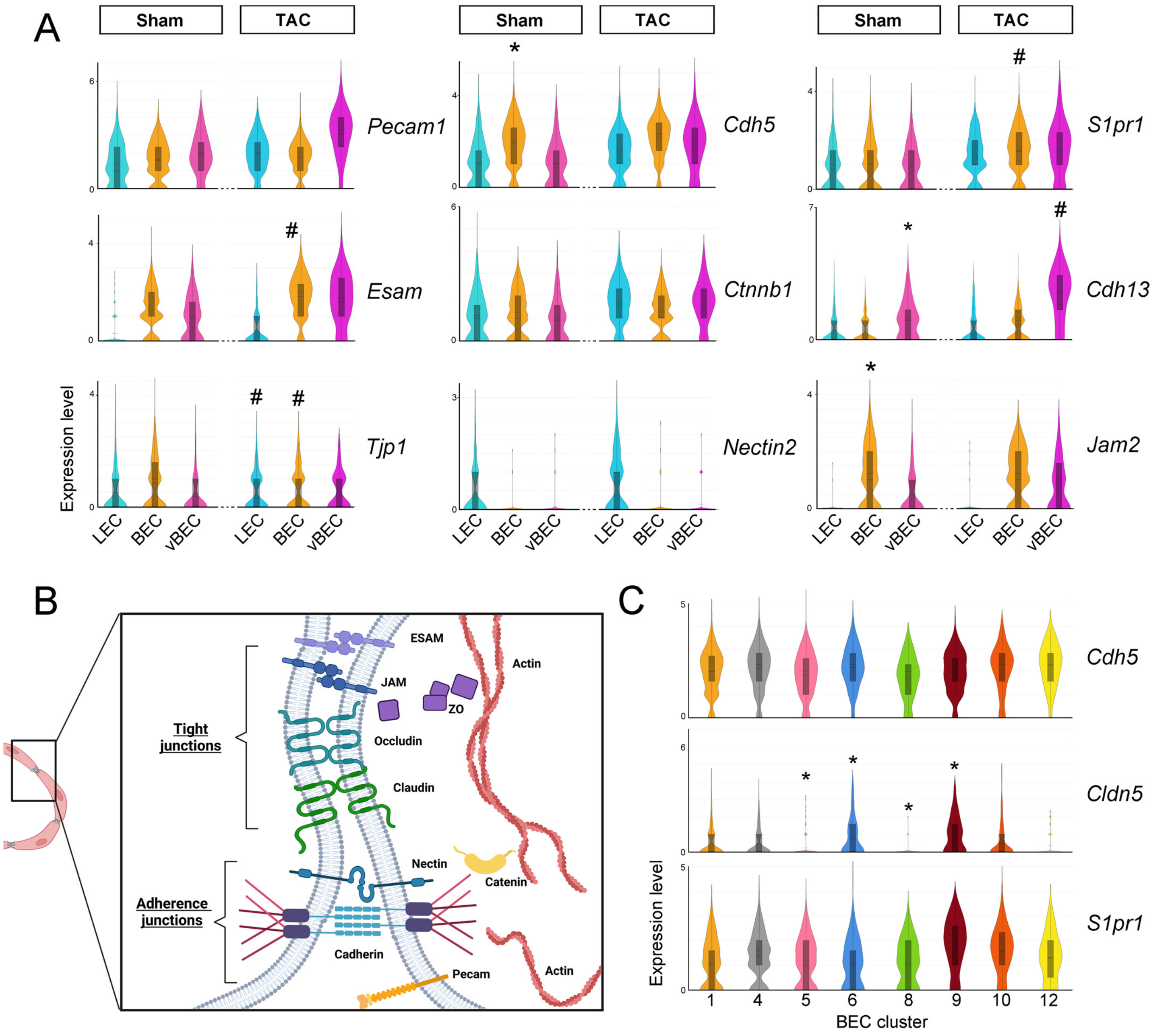

Figure EV3. EC cluster differences in key genes involved in vascular barrier regulation.

(**A**) Examples of expression levels (*Log2 normalized read counts*) of genes involved in adherence junctions and tight junctions in ECs from healthy ($n = 20$) and post-TAC ($n = 10$) BALB/c mice. Clusters significantly enriched for a given gene are denoted (*); whereas genes significantly altered post-TAC are denoted (#). For a full list of EC marker genes, and exact *P* values, see Dataset EV1, and for DEGs post-TAC see Datasets EV4 (LEC), EV5 (BEC), and EV6 (vBEC). (**B**) Schematic overview of junctional assembly. (**C**) Examples of barrier-relevant gene expression levels in healthy cardiac BEC subpopulations. For a list of BEC subpopulation marker genes see Dataset EV2, and for full list of DEGs in BEC subpopulations post-TAC, see Dataset EV8. *Cdh5*, VE-Cadherin; *Cdh13*, T-cadherin; *Ctnnb1*, β-Catenin; *Esam*, Endothelial cell adhesion molecule; *Jam*, Junctional adhesion molecule; *S1pr1*, Sphingosine-1-phosphate receptor 1; *Tjp1*, Tight junction protein-1/ZO-1.

 © The Author(s)

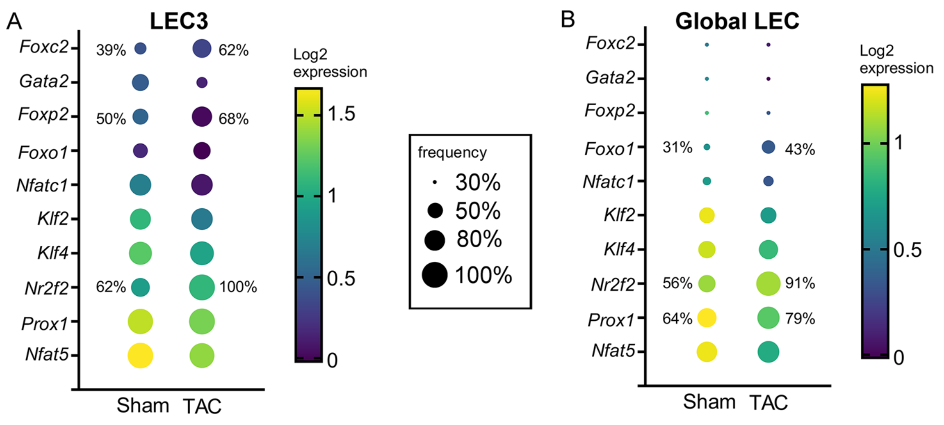

Figure EV4. Frequency of expression of LEC marker genes.

(**A**) Bubble plot illustration of lymphatic transcription factor expression in LEC3 subpopulation in healthy ($n = 20$) and post-TAC ($n = 10$) BALB/c mice (expression levels color-coded as *Log2 normalized read counts*, population frequency of expression coded as bubble size). (**B**) Bubble plot illustration of these genes in the global LEC cluster. For a full list of LEC marker genes, see Dataset EV3.

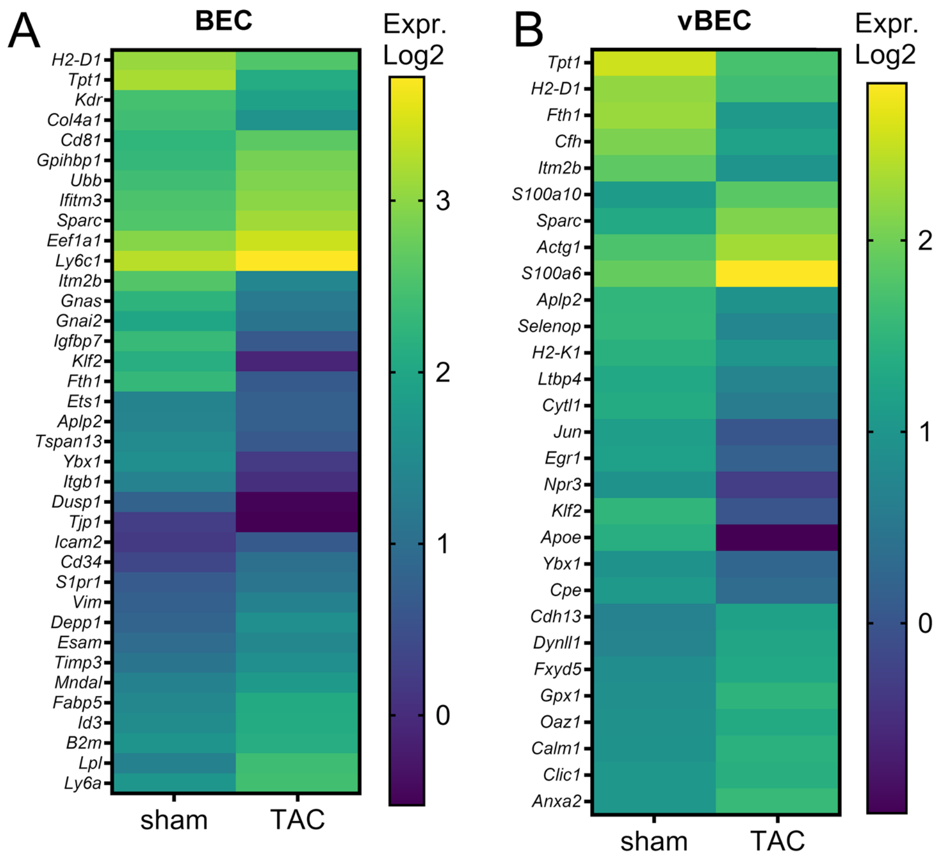

Figure EV5. Differentially expressed genes post-TAC in cardiac BEC and vBEC clusters.

(**A**) Examples of genes differentially expressed post-TAC (*Log2 normalized read counts*) in cardiac BECs in BALB/c mice. (**B**) Examples of genes differentially expressed post-TAC in vBECs. For a full list of DEGs see Dataset EV6 and EV7, respectively.

© The Author(s)

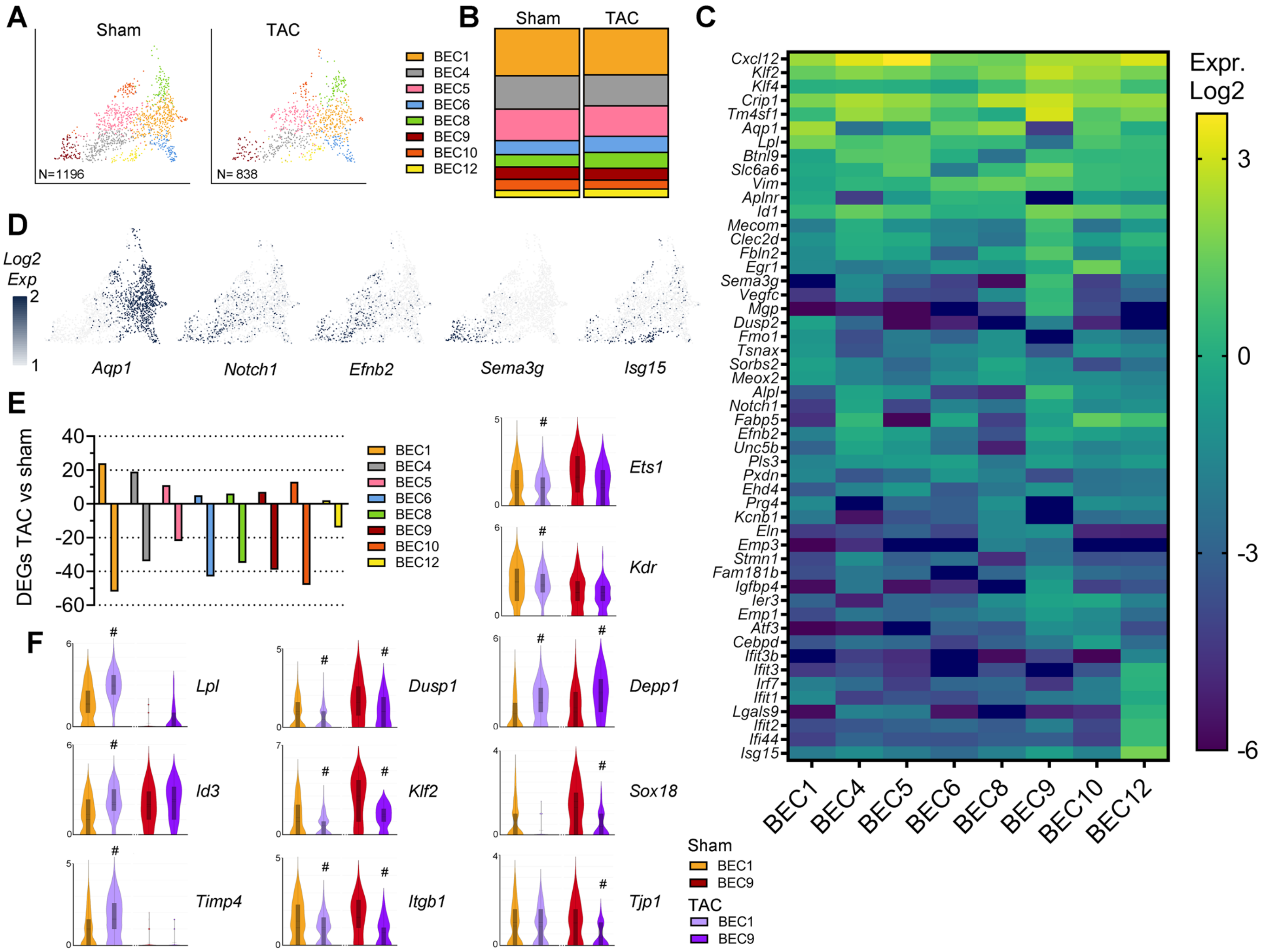

Figure EV6. Subpopulation analyses of cardiac BECs post-TAC in Balb/c.

(**A**) Visualization of cardiac BEC subpopulations in healthy ($n = 20$) and post-TAC ($n = 10$) BALB/c mice. (**B**) BECs from healthy and TAC-operated mice clustered into 8 subpopulations, with the majoritarian BEC1 cluster representing around 30% of cells in both sham and TAC hearts. (**C**) Examples of significantly enriched marker gene expression levels (*Log2 transformed normalized read counts*) in BEC subpopulations. Capillary gene markers were enriched in clusters BEC1, 5, 6, 8, and 10, while arterial gene markers were enriched in clusters BEC4, 5, 9, and 10, while the rare BEC12 cluster displayed an interferon-type signature. For list of marker genes see Dataset EV2. (**D**) Examples of marker gene expression distribution in the global BEC cluster. (**E**) Quantification of DEGs identified post-TAC in each BEC cluster. (**F**) Examples of gene expression levels in sham (*orange* or *red*) and TAC (*purple*) groups in BEC1 and BEC9 clusters. Significantly altered genes indicated (**#**) for the respective cluster. For a full list of DEGs for BEC clusters, and exact p-values, see Dataset EV8.

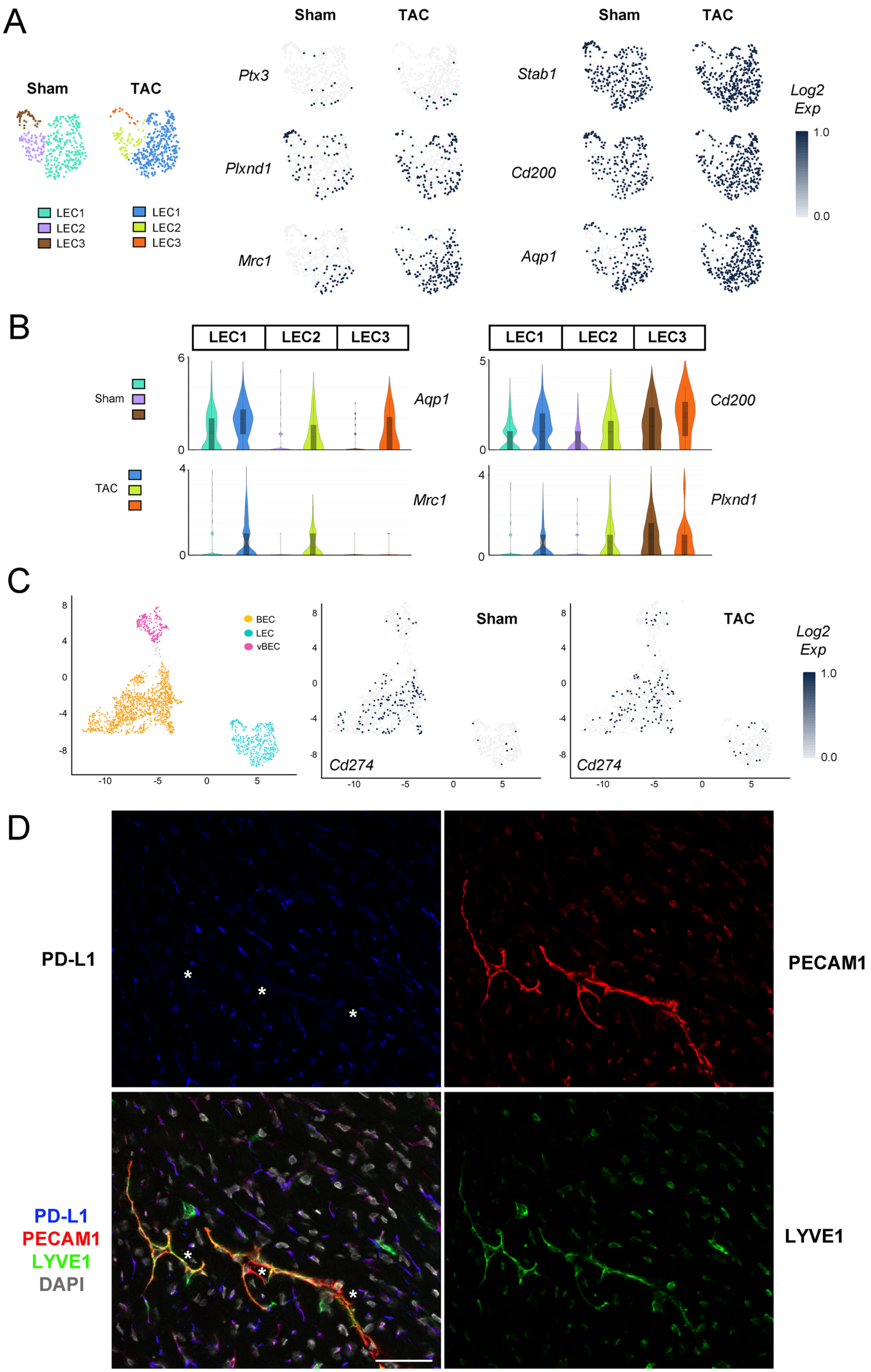
A
Sham
TAC
LEC1
LEC2
LEC3
Ptx3
Plxnd1
Mrc1
Stab1
Cd200
Aqp1
Log2 Exp
1.0
0.0
B
Aqp1
Cd200
Mrc1
Plxnd1
C
BEC
LEC
vBEC
Cd274
D
PD-L1
PECAM1
LYVE1
DAPI

 © The Author(s)

Figure EV7. Cardiac LEC expression of "immune LEC" marker genes.

(**A**) Visualization of cardiac LEC subpopulations in healthy ($n = 20$) and post-TAC ($n = 10$) BALB/c mice, and LEC cluster distribution of marker genes proposed for immune" LEC (iLEC) proposed by Petkova et al. (**B**) Gene expression levels (*Log2 normalized read counts*) of proposed iLEC markers analyzed in LEC subpopulations in healthy and post-TAC Balb/c mice. (**C**) Cardiac EC expression distribution [umap] of *Cd274* (Pdl1) in BECs, vBECs, and LECs in healthy and post-TAC Balb/c mice. (**D**) Vascular PD-L1 protein levels evaluated by immunohistochemistry in cardiac section at 8 weeks post-TAC in Balb/c. PD-L1, *blue*; Lyve1, *green*; CD31, *red*: DAPI, gray. Scale bar 50 µm. *White Asterix*: lymphatics lacking PD-L1 expression. *Bottom left panel*: Purple cells, $CD31^{+}$ blood vessel capillaries expressing Pd-L1; Green cells, $Lyve1^{+}$ $CD31^{neg}$ macrophages.

© The Author(s)

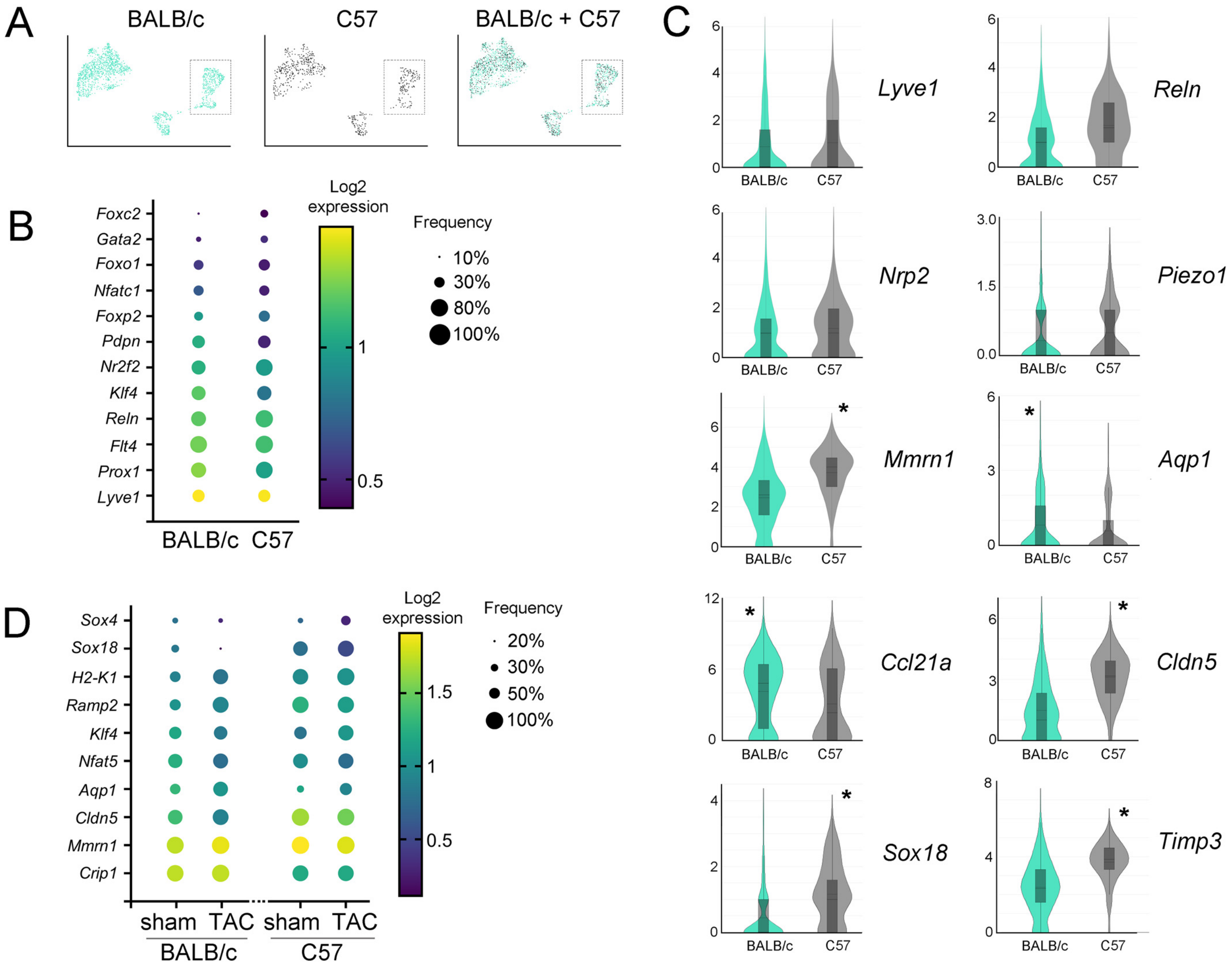

Figure EV8. Comparing LEC markers between strains.

(**A**) UMAP clustering of cardiac vascular EC populations in BALB/c ($N = 1814$ transcriptomes, *blue*) and C57 ($N = 615$, *gray*) from healthy mice ($n = 10$–20 mice/group). The LEC populations are highlighted by dashed lines. (**B**) Comparison of cardiac LEC expression of key lymphatic genes (expression levels color-coded as *Log2 normalized read counts*, population frequency of expression coded as bubble size) in BALB/c vs C57 healthy mice. (**C**) Examples of expression levels (*Log2 normalized read counts*) of key lymphatic markers, including some differentially expressed between BALB/c and C57 healthy mice (indicated by *). For a full list of DEGs, and their associated exact p-values, distinguishing the two strains see Dataset EV10. (**D**) Comparison of cardiac LEC expression of key lymphatic genes (expression level color-coded as *Log2 normalized read counts*, population frequency of expression coded as bubble size) in BALB/c vs C57 healthy or post-TAC mice. Of note, while these genes were not significantly altered post-TAC in C57 mice, many were significantly changed post-TAC in BALB/c (e.g. *Ccl21a*, *Lyve1*, *Klf4, Nfat5, Cldn5*).

 © The Author(s)

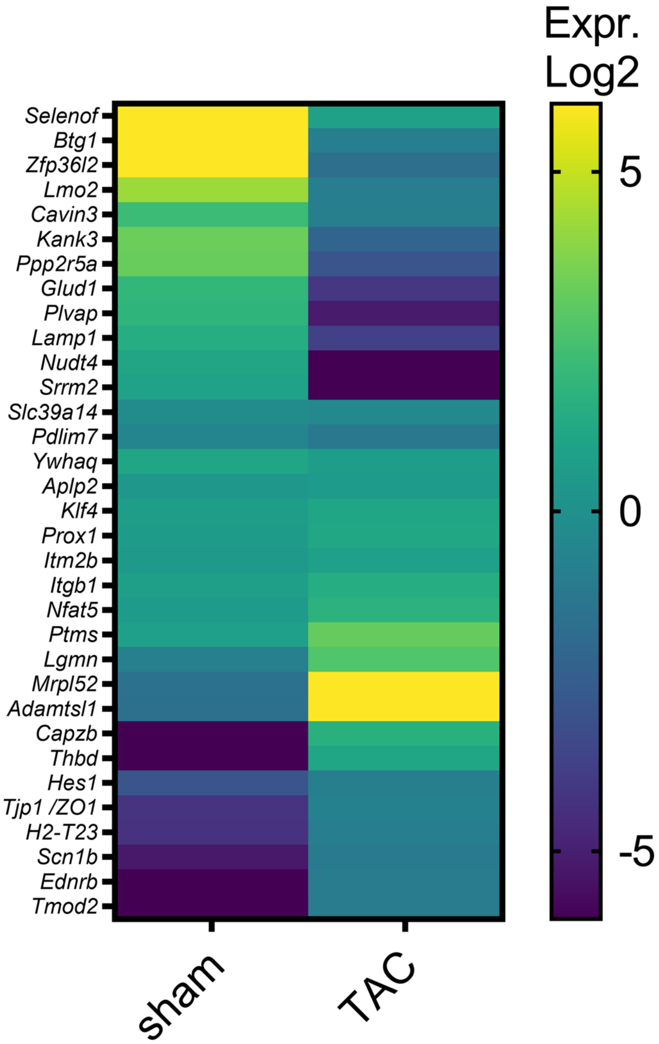

Figure EV9. Differentially-expressed genes in cardiac LECs post-TAC shared with IL1β-stimulated LECs.

Examples of expression levels [*Log2 normalized read counts per cluster*] of genes significantly altered post-TAC in BALB/c mice. These genes were all similarly altered in vitro in IL1β-treated human LECs. For a list of all DEGs in human LECs, see Dataset EV12.
